# Correlation of clinical and histopathological findings in abnormal uterine bleeding

**DOI:** 10.6026/973206300211992

**Published:** 2025-07-31

**Authors:** Priyanka Roy, Sonal Singh, Sangeeta Rai

**Affiliations:** 1 Department of Obstetrics and Gynaecology, Institute of Medical Sciences - Banaras Hindu University, Varanasi, Uttar Pradesh, India

**Keywords:** Abnormal uterine bleeding, endometrial biopsy, histopathology, menorrhagia, endometrial hyperplasia

## Abstract

Abnormal uterine bleeding (AUB) greatly affects female's health condition and thus proper diagnosis is required. A clinico-histopathological correlation was done in this cross-sectional study of 120 women (25 55 yrs) in IMS-BHU. The most frequent symptom was menorrhagia and the most frequent histological finding is proliferative endometrium. There was a high association between age and histopathological trends (p=0.02). Thus, histopathology is necessary in the diagnosis of AUB and in the effective management.

## Background:

Abnormal uterine bleeding (AUB) is a commonly encountered gynecological complaint affecting women across all age groups, particularly during the peri-menopausal period. It refers to bleeding from the uterine corpus that is irregular in volume, duration, or frequency and not associated with regular menstruation [1]. AUB significantly impacts a woman's physical health, emotional well-being and overall quality of life, contributing to a substantial number of outpatient visits and hospital admissions in gynecology departments worldwide [2, 3]. The etiologies of AUB are diverse, ranging from hormonal imbalances to structural abnormalities such as fibroids, polyps, and malignancies. To standardize the diagnosis, the International Federation of Gynecology and Obstetrics (FIGO) introduced the PALM-COEIN classification, which categorizes the causes into structural (PALM: Polyp, Adenomyosis, Leiomyoma, Malignancy and hyperplasia) and non-structural (COEIN: Coagulopathy, Ovulatory dysfunction, Endometrial, Iatrogenic, and Not otherwise classified) components [4]. However, clinical assessment alone often fails to distinguish benign from potentially serious pathologies. Histopathological evaluation of endometrial tissue is considered the gold standard for identifying the underlying cause of AUB [5], as it confirms clinical suspicions, informs treatment strategies, and enables early detection of premalignant or malignant changes [6]. Endometrial sampling via dilatation and curettage or pipelle biopsy is particularly informative in women over 40 years or those with risk factors for endometrial carcinoma [7]. Therefore, it is of interest to describe the clinico-histopathological correlation in women presenting with abnormal uterine bleeding for improved diagnostic accuracy and management.

## Materials and Methods:

This hospital-based cross-sectional study was conducted in the Department of Obstetrics and Gynaecology, Institute of Medical Sciences - Banaras Hindu University, Varanasi, Uttar Pradesh, India. Over a period of one year, Ethical clearance was obtained from the Institutional Ethics Committee prior to the commencement of the study. Informed consent was obtained from all participants. A total of 120 women aged between 25 and 55 years who presented with complaints of abnormal uterine bleeding (AUB) were included. Patients were selected using a consecutive sampling technique. Women with pregnancy-related bleeding, known bleeding disorders, or on anticoagulant therapy was excluded from the study. A detailed history was recorded for each participant, including age, menstrual history, parity, and associated symptoms. General physical and gynecological examinations were performed. Routine investigations such as hemoglobin levels, thyroid profile, and pelvic ultrasonography were done to rule out other possible causes. All patients underwent endometrial sampling either by pipelle biopsy or dilatation and curettage (D & C), depending on the clinical indication. The endometrial specimens were fixed in 10% neutral buffered formalin, processed, and embedded in paraffin. Sections of 4-5 µm thickness were stained with hematoxylin and eosin (H & E) and examined under a light microscope by an experienced pathologist. The histopathological findings were categorized into normal (proliferative or secretory phase endometrium) and abnormal (hyperplasia, atrophy, polyp, chronic endometritis, or malignancy). The clinical diagnosis and histopathological findings were compared and analyzed statistically. Data were entered into Microsoft Excel and analyzed using SPSS software version XX. The Chi-square test was used to assess the correlation between clinical and histopathological findings. A p-value < 0.05 was considered statistically significant.

## Results:

A total of 120 women presenting with abnormal uterine bleeding were enrolled in the study. The age distribution showed that the majority of patients (42.5%) were in the age group of 41-50 years, followed by 31-40 years (30.8%) (Table 1 - see PDF). The most common clinical pattern of bleeding observed was menorrhagia, seen in 54 patients (45%), followed by metrorrhagia in 30 patients (25%), Polymenorrhea in 18 (15%) and postmenopausal bleeding in 18 patients (15%) (Table 2 - see PDF). Histopathological evaluation of endometrial samples revealed that proliferative endometrium was the most common pattern, observed in 42 cases (35%), followed by secretory endometrium in 30 (25%), simple hyperplasia without atypia in 18 (15%), and endometrial polyp in 12 (10%). Other findings included atrophic endometrium in 10 (8.3%), chronic endometritis in 6 (5%), and endometrial carcinoma in 2 cases (1.7%) (Table 3 - see PDF). A statistically significant association was found between clinical diagnosis and histopathological findings (p = 0.03), indicating a good correlation between clinical presentation and underlying pathology. The majority of postmenopausal women with bleeding were found to have atrophic endometrium or hyperplastic changes (Table 4 - see PDF).

## Discussion:

Abnormal uterine bleeding (AUB) remains a frequent gynecological concern, particularly among women in the perimenopausal age group. In the present study, the majority of women presenting with AUB belonged to the age group of 41-50 years, consistent with findings from earlier studies [1, 2]. Hormonal fluctuations during this transitional period are often responsible for endometrial instability and irregular shedding. Menorrhagia was identified as the most prevalent pattern of bleeding in this study, followed by metrorrhagia and polymenorrhea. These findings are comparable to those reported by Mishra *et al.* [3] and Taludkar *et al.* [4], who also found menorrhagia to be the leading symptom of AUB. The variations in clinical patterns highlight the importance of individualized diagnostic approaches. Histopathologically, the most common finding was proliferative endometrium (35%), followed by secretory endometrium (25%), and simple hyperplasia without atypia (15%). These results align with studies conducted by Jose *et al.* [5] and Gothwal *et al.* [6], who reported similar distributions. Proliferative endometrium in AUB cases may reflect anovulatory cycles, particularly in perimenopausal women, where the hormonal imbalance often results in unopposed estrogen stimulation [7]. Endometrial hyperplasia without atypia was observed in 15% of cases, which is significant because, if untreated, it can progress to carcinoma in a subset of patients [8]. This underscores the necessity of histopathological evaluation even in clinically benign cases. Only 1.7% of patients in our study had endometrial carcinoma, comparable to the low prevalence reported in other Indian and international studies [9, 10]. Atrophic endometrium was found in 8.3% of women, predominantly in those presenting with postmenopausal bleeding. Similar findings have been documented by Damle *et al.* [11], emphasizing that atrophy is a common cause of bleeding in postmenopausal women and often benign in nature. Chronic endometritis, found in 5% of cases, often presents with nonspecific bleeding symptoms and is commonly associated with lower genital tract infections or retained products of conception [12]. The PALM-COEIN classification proposed by FIGO has streamlined the diagnostic process for AUB by segregating structural from non-structural causes [13]. However, our study reinforces that histopathological analysis remains a cornerstone in determining the exact etiology and guiding appropriate therapy. Studies by Martinez *et al.* [14] and Nguyen *et al.* [15] have also highlighted the role of tissue sampling in improving diagnostic accuracy and ensuring early detection of premalignant lesions. In our study, a statistically significant correlation (p = 0.03) was observed between clinical patterns and histopathological findings, suggesting that while clinical evaluation is useful, it cannot replace histopathological confirmation. This is particularly vital in differentiating benign conditions like polyps or hyperplasia from malignancies, which require more aggressive intervention. The PALM-COEIN classification system has become integral to the standardized diagnosis and management of abnormal uterine bleeding (AUB), particularly in reproductive and perimenopausal women [16]. Among the various presentations, heavy menstrual bleeding and ovulatory dysfunction are most frequently reported, while leiomyomas and adenomyosis are common structural causes identified through imaging and histopathology [17]. Effective patient care hinges on a strong clinicopathological correlation, emphasizing the importance of endometrial tissue sampling-especially in women over 45-to exclude malignancy and guide individualized treatment strategies. Histopathological evaluation plays a critical role in confirming clinical diagnoses of abnormal uterine bleeding (AUB), especially in reproductive and perimenopausal women [18]. Studies consistently highlight a strong clinico-histopathological correlation in cases of AUB, emphasizing the importance of endometrial sampling in guiding treatment strategies, including decisions regarding hysterectomy [19]. Across different tertiary care centers in India, proliferative endometrium and endometrial hyperplasia emerge as the most common histopathological patterns associated with AUB, underlining the need for routine histological screening to rule out premalignant or malignant conditions [20].

## Conclusion:

Abnormal uterine bleeding in perimenopausal women presents with varied clinical patterns and histopathological evaluation remains essential for accurate diagnosis. The strong correlation between clinical presentation and endometrial histology highlights the importance of combining both approaches for effective patient management and timely identification of premalignant or malignant conditions.
